# Development and Evaluation of a Stable Oil-in-Water Emulsion with High Ostrich Oil Concentration for Skincare Applications

**DOI:** 10.3390/molecules29050982

**Published:** 2024-02-23

**Authors:** Juthaporn Ponphaiboon, Sontaya Limmatvapirat, Chutima Limmatvapirat

**Affiliations:** 1 Department of Industrial Pharmacy, Faculty of Pharmacy, Silpakorn University, Nakhon Pathom 73000, Thailand; augusto_sc@hotmail.co.th (J.P.); limmatvapirat_s@su.ac.th (S.L.); 2 Natural Products Research Center (NPRC), Faculty of Pharmacy, Silpakorn University, Nakhon Pathom 73000, Thailand

**Keywords:** ostrich oil, emulsion, emulsifier, hydrophile–lipophile balance, antibacterial activity, antioxidant activity, cytotoxicity, stability

## Abstract

This study investigates the development of an oil-in-water (O/W) emulsion enriched with a high concentration of ostrich oil, recognized for its abundant content of oleic acid (34.60 ± 0.01%), tailored for skincare applications. Using Span and Tween emulsifiers, we formulated an optimized emulsion with 20% *w*/*w* ostrich oil and a 15% *w*/*w* blend of Span 20 and Tween 80. This formulation, achieved via homogenization at 3800 rpm for 5 min, yielded the smallest droplet size (5.01 ± 0.43 μm) alongside an appropriate zeta potential (−32.22 mV). Our investigation into the influence of Span and Tween concentrations, types, and ratios on the stability of 20% *w*/*w* ostrich oil emulsions, maintaining a hydrophile–lipophile balance (HLB) of 5.5, consistently demonstrated the superior stability of the optimized emulsion across various formulations. Cytotoxicity assessments on human dermal fibroblasts affirmed the safety of the emulsion. Notably, the emulsion exhibited a 52.20 ± 2.01% inhibition of linoleic acid oxidation, surpassing the 44.70 ± 1.94% inhibition observed for ostrich oil alone. Moreover, it demonstrated a superior inhibitory zone against *Staphylococcus aureus* (12.32 ± 0.19 mm), compared to the 6.12 ± 0.15 mm observed for ostrich oil alone, highlighting its enhanced antioxidant and antibacterial properties and strengthening its potential for skincare applications. The optimized emulsion also demonstrates the release of 78.16 ± 1.22% of oleic acid across the cellulose acetate membrane after 180 min of study time. This successful release of oleic acid further enhances the overall efficacy and versatility of the optimized emulsion. Stability assessments, conducted over 6 months at different temperatures (4 °C, 25 °C, 45 °C), confirmed the emulsion’s sustained physicochemical and microbial stability, supporting its promise for topical applications. Despite minor fluctuations in acid values (AV) and peroxide values (PV), the results remained within the acceptable limits. This research elucidates the crucial role of emulsification in optimizing the efficacy and stability of ostrich oil in skincare formulations, providing valuable insights for practical applications where stability is paramount.

## 1. Introduction

Ostrich oil is derived from the adipose tissues of the ostrich bird (*Struthio camelus*). The process that converts these adipose tissues into high-quality crude oil is known as low-temperature wet rendering [1,2,3]. The quality of the oil depends on the temperature and duration of the heating process [3]. Ostrich oil primarily consists of triglycerides and essential fatty acids, particularly α-linolenic acid (omega-3), linoleic acid (omega-6), and oleic acid (omega-9) [4,5]. It has been discovered that these essential fatty acids prevent cardiovascular disease and regulate brain and nervous system function [6]. Omega-3 and omega-6 assume pivotal roles in human health across all life stages, including developmental, maturation, and aging phases [7]. Additionally, the antioxidant, antibacterial, and anti-inflammatory activities of ostrich oil appear to be attributed to minor components such as carotenoids, tocopherol, and flavones [8]. Conventionally employed in traditional medicine, ostrich oil has exhibited anti-inflammatory characteristics that are advantageous in the treatment of ailments including contact dermatitis and eczema. Eltom et al. [9] reported that the application of the isolated topical γ-lactone (5-hexyl-3H-furan-2-one) derived from ostrich oil significantly mitigated the paw edema induced by formalin in rodents. In addition, it was observed that the nano-emulsion comprising 1% *w*/*w* ostrich oil demonstrated more pronounced anti-inflammatory effects in rodent models of carrageenan-induced inflammation than ostrich oil in its pure form [10]. Wound healing applications have made use of ostrich oil, which is recognized for its antimicrobial and anti-inflammatory properties [11]. A study was conducted to assess the prospective wound healing effectiveness of an ointment containing 2% *w*/*w* ostrich oil on mice that had excisional wounds infected with *Pseudomonas aeruginosa* and *Staphylococcus aureus*. In contrast to the ointment base, the ointments containing 2% *w*/*w* ostrich oil exhibited a diminished bacterial count and stimulated the process of wound healing through the mitigation of inflammation, the promotion of fibroblast proliferation, and the enhancement of collagen deposition [12].

However, ostrich oil faces a significant challenge due to its susceptibility to oxidative and hydrolytic rancidity, leading to undesirable odors and colors [13]. To enhance the stability of ostrich oil, innovative oil-in-water (O/W) emulsions have been developed. These emulsions provide several advantages, such as reducing the perception of greasiness and minimizing the occurrence of oil rancidity. The role of emulsifiers is pivotal, as they create a protective film around oil droplets, effectively shielding them from the detrimental effects of oxygen and light exposure [14]. Additionally, prior studies have predominantly focused on topical preparations containing only 1–2% *w*/*w* ostrich oil [10,12]. Thus, the development of emulsion formulations with high concentrations of ostrich oil poses an intriguing challenge.

The formation of oil-in-water (O/W) emulsions relies on emulsifiers that adsorb to the oil–water interface during homogenization, effectively reducing surface tension and preventing undesirable flocculation and coalescence of oil droplets through steric or electrostatic repulsions [15]. Tweens and Spans, belonging to classes of surfactants, are frequently employed together to establish stable emulsions. Tweens possess hydrophilic properties, while Spans exhibit lipophilic characteristics. Their synergistic application ensures the comprehensive coverage of both emulsion phases, contributing to overall stability. The emulsifiers’ amphiphilic nature, combined with their ability to diminish interfacial tension, form micelles, prevent coalescence, and enhance dispersion, plays a crucial role in the effective creation and maintenance of stable emulsions [14].

The objectives of this study were to assess the feasibility of producing an O/W emulsion with a high concentration of ostrich oil, intended to highlight antioxidant and antimicrobial properties. The anticipated benefits cover a diverse range of effects, including antioxidant, anti-scratch, and protective properties.

## 2. Results

### 2.1. Preparation and Evaluation of Ostrich Oil

The fatty acid composition of ostrich oil, obtained through the low-temperature rendering method, was analyzed using the GC-FID technique. Table 1 provides information on the retention time and linearity of FAME standards. Methyl heptadecanoate, serving as an internal standard, exhibited a retention time of 9.1 min. Regression analysis demonstrated correlation coefficients (r^2^) for all FAME standards ranging from 0.9998 to 0.9999, signifying a perfect linear relationship. Upon examination of the fatty acid composition, the three most prevalent fatty acids in ostrich oil were oleic acid (omega-9) at 34.60 ± 0.01%, palmitic acid at 28.42 ± 0.05%, and linoleic acid (omega-6) at 27.73 ± 0.01%. Additionally, stearic acid was identified at 5.07 ± 0.05%, α-linolenic acid (omega-3) at 3.02 ± 0.00%, myristic acid at 0.93 ± 0.01%, and lauric acid at 0.23 ± 0.01%.

Ostrich oil is well regarded for its nutritional value, primarily attributed to its high levels of omega-3, omega-6, and omega-9 fatty acids. This composition positions ostrich oil as a beneficial adjunct therapy for various skin conditions, offering advantages such as moisturizing properties, support for skin barrier function, anti-inflammatory effects, and wound healing capabilities [16,17]. Furthermore, palmitic acid has been identified for its antibacterial properties [18], while stearic acid exhibits anti-inflammatory properties for the skin [19]. As a result, ostrich oil is widely recognized as an excellent source of nutrients that contribute to overall skin health.

In line with the Codex Standard for Named Animal Fats (CODEX STAN 211-1999) established by the Food and Agriculture Organization of the United Nations (FAO) and the World Health Organization (WHO) [20], the ostrich oil exhibited AV and PV of 0.10 ± 0.00 mg KOH/g oil and 2.53 ± 0.12 mEq O_2_/kg oil, respectively. These values were well within the allowed limits of 2 mg KOH/g oil and 10 mEq O_2_/kg oil. The concentrations of Fe and Cu were measured at 0.803 ± 0.148 mg/kg and 0.002 ± 0.001 mg/kg, respectively. Additionally, As and Pb were not detected, all falling comfortably within the highest allowable concentrations of 1.5 mg/kg for Fe, 0.4 mg/kg for Cu, 0.1 mg/kg for As, and 0.1 mg/kg for Pb. The microbial limit test revealed that neither TAMC nor TYMC were present in the oil sample. *S. aureus*, *P. aeruginosa*, *Clostridium* spp., and *C. albicans* contamination in 1 g of any sample was not detected during pathogen investigation. The outcomes indicate that the prepared ostrich oil was of exceptional quality and safe for human use.

Ostrich oil at a high dose (360 mg/mL, the maximal solubility in the test medium) exhibited no anti-inflammatory activity. In a previous report, ostrich oil was found to lack anti-inflammatory activity in 15-day-treated mice at doses of 100 or 500 mg/kg/day [21]. Additional anti-inflammatory experiments are required to investigate its mechanism and potential efficacy.

The IC_50_ of DPPH and the percentage of inhibition of linoleic acid oxidation (Day 5) for ostrich oil were 39.92 ± 1.51 mg/mL and 44.70 ± 1.94%, respectively, while those for Trolox were 0.0043 ± 0.0001 mg/mL and 58.20 ± 5.18%, respectively. The maximal percent inhibition of linoleic acid oxidation by ostrich oil and Trolox occurred on day 5 and lasted until day 7; 41.12 ± 1.83 and 55.82 ± 2.03%, respectively. Therefore, the results demonstrated that ostrich oil possesses a potent antioxidant activity and a long duration of linoleic acid oxidation inhibition.

Based on the analysis results mentioned earlier, which encompassed the fatty acid profile, antioxidant activity, microbial contamination, and heavy metal contents, it is possible to deduce that the ostrich oil generated via the low-temperature wet rendering method was appropriate for the formulation of an emulsion intended for topical application on the skin.

### 2.2. Formulation and Evaluation of Emulsion Containing Ostrich Oil

#### 2.2.1. Determination of Required Ostrich Oil HLB

**Figure 1:**
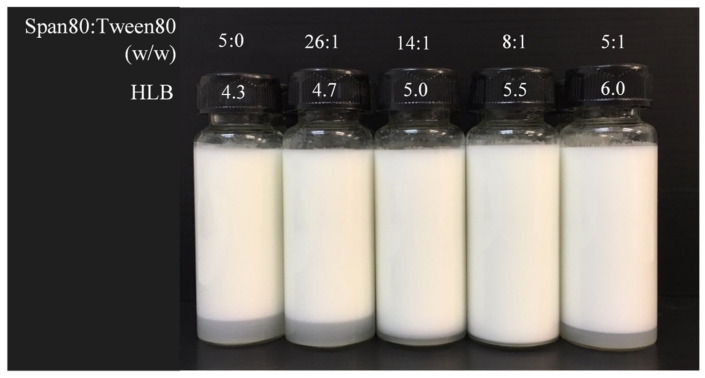
The physical appearances of emulsions with varying HLB values containing 20% *w*/*w* ostrich oil and 5% *w*/*w* mixed emulsifier of Span 80 and Tween 80 in varied ratios.

The evaluation of ostrich oil HLB values was conducted using the beaker method to prepare O/W emulsions. These emulsions consisted of 20% *w*/*w* ostrich oil and 5% *w*/*w* emulsifiers, comprising different ratios of Span 80 and Tween 80, as shown in Table 2. The stability of all of the prepared emulsions was assessed by storing them in cylindrical glass vials at room temperature for 24 h, followed by visual observation for any separation between the aqueous and oil phases, as depicted in Figure 1. The findings revealed that the emulsion with an HLB value of 5.5 displayed the highest stability, showing no phase separation (creaming index of 0.00%) (Table 2). Based on these results, it can be inferred that the optimal HLB value for ostrich oil is 5.5, indicating that this particular HLB value is essential for achieving the desired emulsion stability.

HLB values are used to characterize the surfactant’s balance between hydrophilic (water-attracting) and lipophilic (oil-attracting) properties. For the preparation of O/W emulsions, higher HLB values are generally preferred because they indicate a higher degree of hydrophilicity, making the surfactant more effective at stabilizing water droplets in an oil phase [22]. The optimal HLB value depends on the specific formulation and the types of oils and emulsifiers used. The exact HLB value will depend on the specific requirements of the formulation, the nature of the oils and other ingredients used, and the desired characteristics of the final product. It is important to note that formulating emulsions involves a combination of emulsifiers and possibly co-emulsifiers to achieve the desired stability and texture. Experimentation may be necessary to find the optimal HLB value for a particular emulsion formulation.

The suitability of a particular HLB value for preparing an O/W emulsion depends on various factors, including the specific properties of the oil and the emulsifiers used. In the case of ostrich oil, it appears that an HLB value of 5.5 is considered appropriate for preparing an O/W emulsion. Ostrich oil is known to contain a mixture of fatty acids, and its composition can influence its behavior in emulsion systems. Emulsifiers are chosen based on their HLB values to achieve stability and proper emulsification of the oil phase in the water phase. An HLB value of 5.5 suggests that the emulsifier or emulsifier blend used has a moderate affinity for both water and oil, making it suitable for creating an O/W emulsion with ostrich oil. It is important to note that the specific formulation, the desired characteristics of the final product, and the interaction between the emulsifier and ostrich oil will all play a role in determining the optimal HLB value. Experimentation and testing may be required to finetune the formulation and confirm that an HLB of 5.5 is indeed suitable for achieving the desired stability and characteristics in the ostrich oil O/W emulsion.

#### 2.2.2. Effects of the Type and Concentration of Mixed Emulsifiers on the Properties of Emulsions

**Figure 2:**
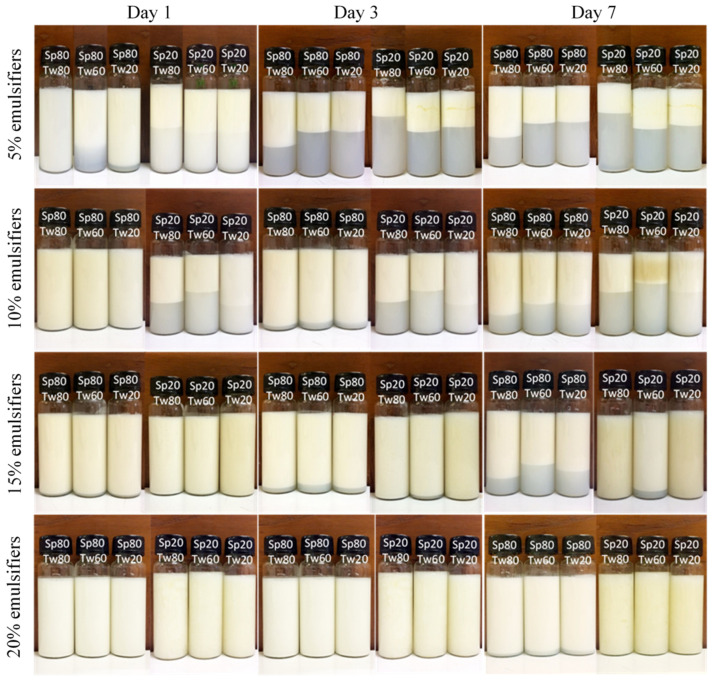
Visual characteristics of emulsions containing 20% *w*/*w* ostrich oil and a 5, 10, 15, or 20% *w*/*w* emulsifier mixture of Span (Sp) and Tween (Tw) on days 1, 3, and 7.

When an emulsifier with a particular HLB value stabilizes an emulsion containing a particular type of oil, it is generally assumed that another emulsifier with the same HLB value will have a similar emulsifying effect [23,24,25]. Therefore, each variety of oil requires an emulsifier system with a unique HLB number, which corresponds to the required HLB of the oil phase. In this study, O/W emulsions with an HLB of 5.5 were prepared with 20% *w*/*w* ostrich oil and different ratios of 5, 10, 15, or 20% *w*/*w* mixed Span and Tween emulsifiers (Table 3). After preparing the O/W emulsions, they were stored at room temperature for 1, 3, and 7 days. Subsequently, their physical characteristics were assessed visually (Figure 2), and their CI percentages were calculated (Table 4). The viscosity, droplet size, zeta potential, and photomicrographs of emulsions were evaluated.

This study focuses on the effects of different Span and Tween concentrations, types, and ratios on the stability of 20% *w*/*w* ostrich oil emulsions with a target HLB value of 5.5. The Span–Tween ratio listed in Table 3 was derived from the experimental data in Table 2. It was determined that Span 80–Tween 80, at a weight ratio of 4.44:0.56 (or 8:1), resulted in an emulsion with an HLB value of 5.5, leading to excellent stability (CI equal to 0.00%). The emulsifier ratios provided in Table 3 were calculated by considering the weight of each emulsifier relative to its specific HLB value, aiming to achieve an emulsion with an overall HLB value of 5.5. The formulation of emulsions with an HLB of 5.5, comprising 20% *w*/*w* ostrich oil, and varying concentrations (5%, 10%, 15%, or 20% *w*/*w*) of combined Span and Tween emulsifiers according to the formulas in Table 3, was carried out to evaluate the suitability of ostrich oil and emulsifier weights. The experimental results revealed that the emulsion stabilized with a 15% *w*/*w* mixture of Span 20 and Tween 80 exhibited the highest stability among all of the formulations tested, with desirable properties in terms of creaming indices (Table 4).

#### 2.2.3. Viscosity

**Figure 3:**
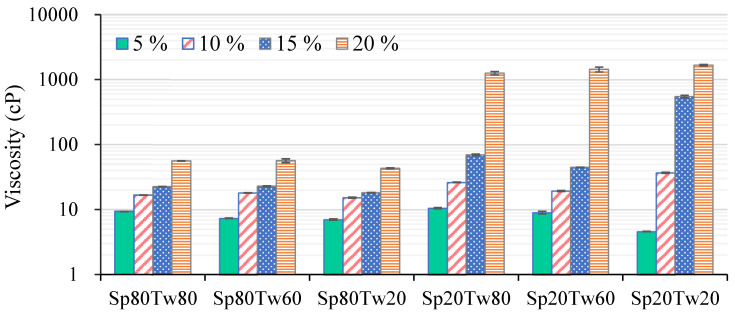
Viscosities of emulsions containing 20% *w*/*w* ostrich oil and 5, 10, 15, or 20% *w*/*w* Span (Sp) and Tween (Tw) mixed emulsifiers.

The viscosity profiles of emulsions, featuring 5%, 10%, 15%, and 20% *w*/*w* mixed emulsifiers (Span and Tween), combined with 20% *w*/*w* ostrich oil, are depicted in Figure 3. An observable trend revealed a gradual increase in viscosities as the concentrations of mixed emulsifiers ascended from 5% to 15% *w*/*w*, indicating a positive correlation between emulsifier concentration and viscosity. Subsequently, a pronounced surge in viscosities occurred with 20% *w*/*w* mixed emulsifiers (Span 20 combined with Tween 80, Tween 60, or Tween 20). As previously reported [26], the rise in viscosity can be attributed to the confinement of water molecules within the crosslinking areas of the emulsifiers. This process enhances the hydration of water molecules that are encircling the hydrophilic portions of the emulsifiers. Addressing stability, both emulsifier concentration and type were identified as factors influencing the viscosity and stability of the emulsions [14]. The findings, derived from the creaming index analysis (Table 4) and viscosity measurements (Figure 3), suggested that an optimal concentration for formulating O/W emulsions containing 20% *w*/*w* ostrich oil was 15% *w*/*w* for mixed emulsifiers. Insights into the manner in which blended emulsifiers influence the viscosity and stability of emulsions are gained from these results, highlighting the importance of emulsifier type and concentration when preparing stable emulsions with ostrich oil.

#### 2.2.4. Droplet Size

**Figure 4:**
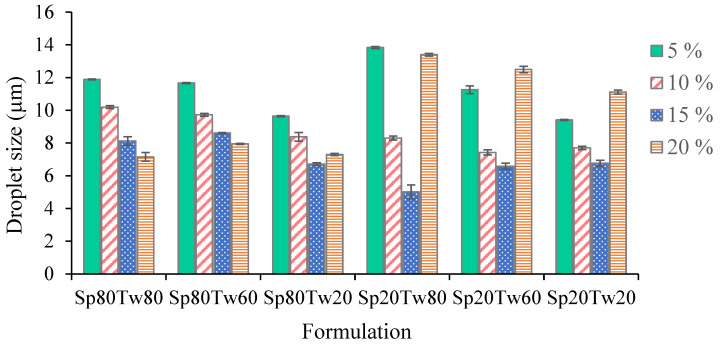
Droplet size of the emulsions comprising 20% *w*/*w* ostrich oil and a 5, 10, 15, or 20% *w*/*w* mixture of Span (Sp) and Tween (Tw).

The droplet size of oil dispersion in emulsions is depicted in Figure 4. The results demonstrate that as the concentrations of the Span and Tween mixture increased from 5% to 15% *w*/*w*, there was a gradual reduction in emulsion droplet size. This phenomenon can be attributed to the increased reduction in surface tension between oil and water, leading to a decrease in interfacial free energy. The emulsifier plays a crucial role in both droplet break up and recoalescence by lowering interfacial tension and preventing immediate recoalescence [27,28]. The emulsion containing 20% *w*/*w* ostrich oil emulsified with a 15% *w*/*w* mixture of Span 20 and Tween 80 exhibited the smallest droplet size (5.01 ± 0.43 μm), as shown in Figure 4. Additionally, this emulsion formulation achieved a creaming index of 0.00% (Table 4), indicating a high level of confidence in the precision of the results. This underscores the effectiveness of the emulsifier mixture in achieving fine droplet sizes and enhancing the stability of the emulsion.

#### 2.2.5. Microscopic Examination

**Figure 5:**
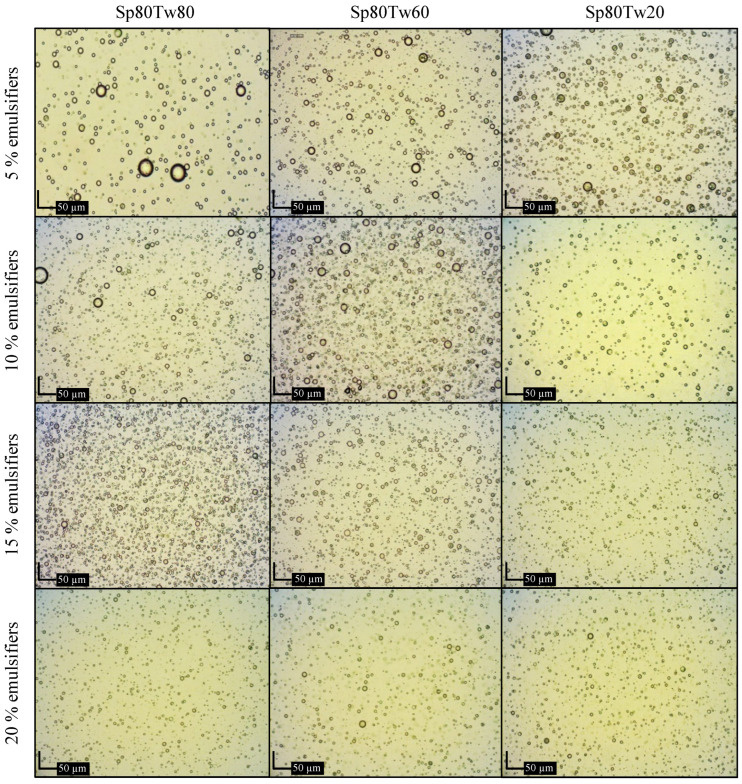
Photomicrographs of emulsions containing 20% *w*/*w* ostrich oil and 5, 10, 15, or 20% *w*/*w* mixtures of Span (Sp) 80 and Tween (Tw) 20, 60, or 80.

**Figure 6:**
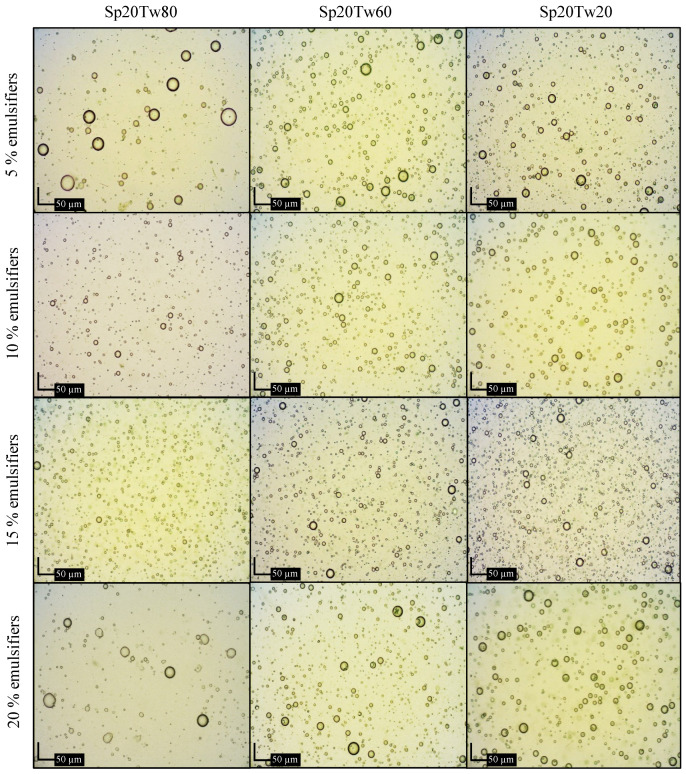
Photomicrographs of emulsions containing 20% *w*/*w* ostrich oil and 5, 10, 15, or 20% *w*/*w* mixtures of Span (Sp) 20 and Tween (Tw) 20, 60, or 80.

Photomicrographs illustrating emulsions containing 20% *w*/*w* ostrich oil and 5%, 10%, 15%, or 20% *w*/*w* mixtures of Span 80 and Tween 20, 60, or 80 are presented in Figure 5. Additionally, Figure 6 displays photomicrographs of emulsions containing 20% *w*/*w* ostrich oil and 5%, 10%, 15%, or 20% *w*/*w* mixtures of Span 20 and Tween 20, 60, or 80. In Figure 5, it is evident that the droplet size of emulsions stabilized with higher concentrations of mixed emulsifiers (15% *w*/*w* and 20% *w*/*w*) is smaller compared to those with lower concentrations (5% *w*/*w* and 10% *w*/*w*). Notably, the emulsion stabilized with 15% *w*/*w* Span 20 and Tween 80 exhibited the smallest droplet size (Figure 6). These findings align with the droplet size information previously discussed in Section 2.2.4. The photomicrographs provide a visual confirmation of the relationship between emulsifier concentration and droplet size, supporting the idea that higher concentrations contribute to smaller droplet sizes. This information enhances our understanding of the emulsion stability achieved through specific emulsifier formulations, particularly emphasizing the efficacy of 15% *w*/*w* Span 20 and Tween 80 in minimizing droplet sizes in the presented emulsion system.

#### 2.2.6. Zeta Potential

**Figure 7:**
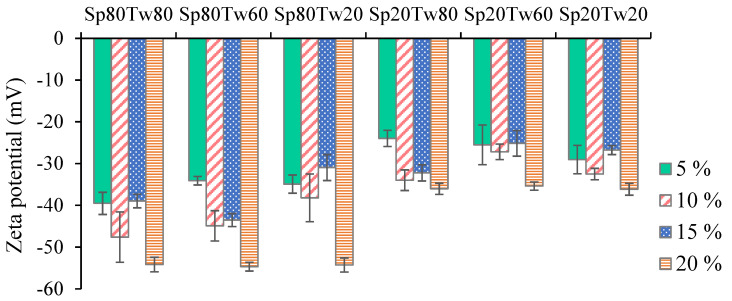
Zeta potential of the emulsions containing 20% *w*/*w* ostrich oil and 5, 10, 15, or 20% *w*/*w* mixtures of Span (Sp) and Tween (Tw).

The zeta potential, representing electric charges on particle surfaces, serves as a crucial parameter for assessing emulsion stability. In this study, the zeta potential values for all emulsions consistently exhibited negative charges. According to previous research [14], a stable emulsion is achieved when the mutual repulsion among emulsion droplets surpasses attractive forces. Examining the zeta potential values in Figure 7, it was observed that the emulsion stabilized with a 5% *w*/*w* concentration of Span 20 and Tween 80 displayed the highest zeta potential value (−23.97 ± 1.94 mV). Conversely, the emulsion stabilized with a 20% *w*/*w* concentration of Span 80 and Tween 60 exhibited the lowest zeta potential value (−4.68 ± 1.05 mV). Notably, negative zeta potential values in the emulsions were attributed to the carboxyl group of fatty acids present in ostrich oil.

For stable emulsions, it is generally recommended that zeta potential values exceed ±30 mV to prevent deflocculation of the emulsion system [29]. In this context, the emulsion stabilized with a 15% *w*/*w* concentration of Span 20 and Tween 80, which showed a creaming index of 0.00% (Table 4), demonstrated a zeta potential value of −32.22 mV (Figure 7). These results emphasize that zeta potential values are influenced by the concentrations, types, and ratios of mixed emulsifiers. The study contributes valuable insight into how manipulating these parameters can impact the stability of emulsions, providing practical considerations for formulating stable emulsion systems.

Zeta potential is an essential determinant of the stability of an emulsion. As depicted in Figure 7, the emulsion produced by combining 15% *w*/*w* Span 20 and Tween 80 demonstrated a notably elevated negative value of −32.22 mV. Strong electrostatic repulsion exists between the dispersed oil particles in the aqueous phase, as indicated by this result. An increase in surface charge contributes to the stability of an emulsion through the promotion of repulsive forces among particles, thereby impeding contraction and coalescence [30]. In general, the zeta potential is utilized to assess the stability of droplets. Zeta potential values exceeding +30 mV or −30 mV indicate a favorable emulsion stability [31]. A comparatively stable system is produced when the repulsive forces of an emulsion outweigh the attractive forces, as indicated by a high zeta potential.

In this section, the influence of concentrations, types, and ratios of Span and Tween on the stability of 20% *w*/*w* ostrich oil emulsions with an HLB of 5.5 was thoroughly investigated, encompassing various physical characteristics, creaming indices, viscosities, droplet sizes, and zeta potentials. The outcomes consistently revealed that the 20% *w*/*w* ostrich oil emulsion stabilized with a 15% *w*/*w* combination of Span 20 and Tween 80 exhibited the highest degree of stability among the formulations. This finding underscores the importance of the chosen emulsifier composition and its concentration in achieving optimal stability for ostrich oil emulsions with the specified HLB.

#### 2.2.7. Stability

To assess stability, samples from the optimized emulsion, comprising 20% *w*/*w* ostrich oil and 15% *w*/*w* mixed emulsifiers (Span 20 and Tween 80), with and without 0.01% *w*/*w* BHT, were subjected to storage at different temperatures: 4 °C, 25 °C, and 45 °C. The evaluation included measurements of AV, PV, phase separation, and microbial contamination at various time points (0, 1, 3, and 6 months). AV and PV served as indicators for assessing the physicochemical stability of the optimized ostrich oil emulsions with and without BHT. An increase in free fatty acids (elevated AV) in samples suggests triglyceride hydrolysis of ostrich oil in the emulsions. Compounds such as free butyric, capric, caprylic, and caproic acids, produced by hydrolysis, are responsible for the rancid flavor of oils. PV, on the other hand, indicates the level of primary oxidation in oils by measuring the hydroperoxides generated during oxidation. Therefore, AV and PV are key parameters associated with the stability of the oil [32].

The Avs of samples with and without BHT stored at 4 °C and samples with BHT at 25 °C did not exhibit significant differences over the 6-month storage period, as indicated in Table 5. During the initial month of storage, Avs of samples without BHT kept at 25 °C showed a slight increase, while those of samples without BHT stored at 45 °C experienced a substantial increase. Following the first month of storage, samples maintained at higher temperatures (25 °C and 45 °C) displayed elevated Avs, with the highest values observed in samples without BHT stored at 45 °C (Table 5). Concerning PVs of samples stored at different temperatures, PVs of samples determined at 4 °C and 25 °C slightly increased, while the values of samples stored at 45 °C exhibited a considerable increase over the 6-month storage period. Notably, samples without BHT stored at 45 °C for 6 months displayed the highest AV and PV. These findings suggest that ostrich oil in emulsions is more susceptible to deterioration and rancidity at higher temperatures in the absence of BHT. In summary, the emulsion of ostrich oil with BHT contributes to increased stability, particularly at elevated temperatures. However, it is noteworthy that the AVs and PVs obtained from the stability testing remained within the allowable limits of 2 mg KOH/g sample and 10 mEq O_2_/kg sample [20]. This suggests that, despite the observed changes, the quality of the ostrich oil emulsions remained within the acceptable standards throughout the storage period. Furthermore, the results of the AVs and PVs in Table 5 showed that the optimized O/W emulsions could maintain the stability of ostrich oil by reducing hydrolysis and oxidation processes.

The phase separation of samples obtained from different storage temperatures was determined using creaming indices expressed as percentages. All emulsion samples, both with and without BHT, kept at 4 °C, 25 °C, and 45 °C for 6 months exhibited creaming indices of 0.00%, indicating excellent physical stability. No microbial contamination by *S. aureus* and *P. aeruginosa* was detected in 1 g of each sample, with or without BHT, over the 6-month period at 4 °C, 25 °C, and 45 °C, aligning with the USP acceptance criteria for the microbiological quality of non-sterile topical dosage forms [33], which specifies the absence of *S. aureus* and *P. aeruginosa* in 1 g of any samples. The TAMC and TYMC of samples with BHT were not detected, while those without BHT gradually increased at all storage temperatures for 1, 3, and 6 months. Particularly, samples without BHT stored at 45 °C exhibited the highest TAMC (50 CFU/g) and TYMC (<10 CFU/g). However, it is important to note that these values remained within the USP acceptance criteria for the microbiological quality of non-sterile topical dosage forms [34], which specify that TAMC and TYMC should not exceed 10^2^ CFU/g and 10^1^ CFU/g, respectively. Thermal stability investigations over the 6-month period indicated that the optimized formulations with BHT remained largely unaffected by a range of climatic conditions, except for occasional instances of phase separation and microbial contamination.

The comprehensive examination of the optimized emulsion’s stability under diverse temperature conditions is crucial for assessing its suitability for real-world applications. By subjecting the emulsion to controlled environments, the study aimed to simulate conditions it might encounter during storage and transportation. The inclusion of ostrich oil, mixed emulsifiers, and BHT in the formulation suggests a complex composition designed for specific performance and stability. BHT, known for its antioxidant properties [34], is commonly used to prevent oxidation and enhance the stability of oil formulations. The emulsifiers, Span 20 and Tween 80, likely contribute to the emulsion’s stability and consistency. This study’s findings, indicating that temperature variations within the specified range did not lead to significant changes in the emulsion’s integrity, are promising. This suggests that the formulation maintains its structural and functional properties even under challenging climatic conditions. Such stability is essential for ensuring the product’s effectiveness and safety over time. It is worth noting that the use of controlled storage conditions and rigorous testing methodologies enhances the reliability of the results. These findings provide valuable insights into the robustness of the emulsion, supporting its potential for practical applications where stability is a critical factor.

#### 2.2.8. Cytotoxicity Assay

An in vitro cytotoxicity assessment was carried out to gauge the safety of the optimized emulsion containing ostrich oil, specifically formulated for topical skin application. Human dermal fibroblasts served as the model organism for assessing potential skin damage. Remarkably, when subjected to the optimized emulsion comprising 20% *w*/*w* ostrich oil and 15% *w*/*w* mixed emulsifiers (Span 20 and Tween 80) at a concentration below 3.2 mg/mL for a duration of up to 24 h, CRL-2076 human dermal fibroblasts exhibited no statistically significant difference in viability compared to the control cells. This suggests a favorable safety profile for the formulation, highlighting its potential suitability for skin application.

#### 2.2.9. Antibacterial Assay

To assess the antibacterial efficacy, the following microorganisms were employed: Gram-positive *S. aureus* ATCC 6538P, Gram-negative *E. coli* DMST 4212, and Gram-negative facultatively anaerobic and aerobic *P. aeruginosa* ATCC 9027. According to Table 6, in the specified sequence, the inhibition zones of the optimized emulsion comprising 20% *w*/*w* ostrich oil and 15% *w*/*w* mixed emulsifiers (Span 20 and Tween 80) measured 12.32 ± 0.19 mm, 6.93 ± 0.18 mm, and 6.3 ± 0.14 mm. In contrast, when tested exclusively on *S. aureus*, ostrich oil demonstrated an inhibitory zone measuring 6.12 ± 0.15 mm. No inhibition zone was observed against *E. coli* or *P. aeruginosa*. Bactericidal effects of lincomycin were detected against all microorganisms during testing, as indicated by inhibitory zones with respective dimensions of 27.00 ± 0.65 mm, 21.03 ± 0.25 mm, and 8.02 ± 0.18 mm.

The results reveal that the optimized emulsion demonstrated antibacterial activity against the tested microorganisms. Upon closer examination, it can be deduced that Gram-positive bacteria exhibit greater susceptibility to the optimized emulsion with ostrich oil. This heightened susceptibility is likely attributable to variations in the cellular wall architecture. Gram-positive bacteria are characterized by a thick peptidoglycan layer and, in some instances, the presence of teichoic acids. In contrast, Gram-negative bacteria possess a thinner peptidoglycan layer surrounded by an outer membrane containing lipopolysaccharides. These structural distinctions have significant implications for bacterial susceptibility to various substances. For instance, the outer membrane of Gram-negative bacteria can confer increased resistance to certain antibiotics or bioactive compounds compared to Gram-positives [35].

Bacteria, including *S. aureus*, *E. coli*, and *P. aeruginosa*, are commonly associated with various infections and are frequently found in diverse environments. The presence of these bacteria in a scratch or wound may indicate a potential risk of infection, as they are recognized pathogens [36]. *S. aureus* is typically present on the skin and mucous membranes and can lead to skin infections, abscesses, and more severe conditions, such as bloodstream infections. Certain strains of *E. coli* have the potential to cause infections, particularly if they contaminate wounds. *P. aeruginosa* is notorious for causing skin and soft tissue infections, especially in wounds and burns. Therefore, it is imperative to seek antibacterial topical products for the treatment of scratches or wounds. However, the appropriate treatment will depend on the severity of the infection and the specific bacteria involved.

The mechanisms of action of ostrich oil against bacteria might involve the disruption of cell membrane integrity [4,5,37,38,39,40,41] and interference with metabolic processes [41]. While the antibacterial mechanisms of fatty acids in ostrich oil remain unknown, these compounds bear resemblance to the bipolar membrane structure of bacteria, characterized by both hydrophilic heads and hydrophobic tails. This similarity suggests that the fatty acids might target bacterial cell membranes, potentially penetrating and disrupting their essential membrane barrier functions [42]. Emulsification might enhance the bioavailability [43,44], stability [45,46], and penetration of ostrich oil [12], thereby potentially augmenting its antibacterial efficacy compared to its non-emulsified form (ostrich oil alone).

#### 2.2.10. Antioxidant Assay

The antioxidant activity of ostrich oil and an optimized emulsion containing 20% *w*/*w* ostrich oil and 15% *w*/*w* mixed emulsifiers (Span 20 and Tween 80) was evaluated by measuring the percentage inhibition of linoleic acid oxidation at a test concentration of 2.4 mg/mL on day 5. The results showed that the percentage inhibition for ostrich oil and the optimized emulsion was 44.70 ± 1.94% and 52.20 ± 2.01%, respectively. For DPPH scavenging activity, ostrich oil and its optimized emulsion exhibited IC_50_ values at concentrations of 39.92 ± 1.51 mg/mL and 23.52 ± 1.09 mg/mL, respectively. In comparison, Trolox (the positive control) showed a percentage inhibition of 58.20 ± 5.18% and a DPPH IC_50_ of 0.0043 ± 0.0001 mg/mL (Table 6). These findings indicate that the antioxidant activity of ostrich oil is enhanced after encapsulation in an O/W emulsion. Notably, the optimized emulsion exhibited a significantly higher percentage inhibition compared to ostrich oil alone. The observed influence of the O/W emulsion on antioxidant activity was found to be statistically significant (*p* < 0.05).

The application of antioxidant activity can be advantageous when it comes to treating injuries and wounds. Antioxidants are compounds that aid in the neutralization of free radicals, which are detrimental molecules capable of inducing oxidative stress and causing cellular damage [47]. Antioxidants have the potential to alleviate inflammation, a physiological reaction that occurs in response to injury but can hinder the recovery process when it becomes excessive [48]. Antioxidants serve to safeguard cells against oxidative damage, thereby facilitating the body’s intrinsic capacity to restore compromised tissues. Scar formation may be mitigated with the assistance of antioxidants, which promote the regeneration of healthy skin cells. Antioxidants have the potential to facilitate the synthesis of collagen, an essential protein that is involved in the structure of the epidermis and the healing of wounds [49].

It is crucial to acknowledge that although antioxidant-rich substances may provide advantages, the principal emphasis in wound care lies in maintaining wound cleanliness, infection protection, and a conducive environment for uncomplicated healing. While topical ostrich oil emulsions featuring antioxidant and antibacterial properties may be regarded as supplementary treatments, it is imperative to seek the advice of a healthcare professional prior to implementing any additional measures, particularly when dealing with severe injuries or infections that require immediate attention.

#### 2.2.11. In Vitro Release Study

The skin permeability for transdermal administration was monitored using Franz diffusion cells. In vitro release studies of the optimized emulsion containing ostrich oil were conducted over 180 min using a cellulose acetate membrane, providing insights into sample diffusion across the skin [50]. Analysis of the fatty acid composition in this study revealed that oleic acid (omega-9) was the most prevalent fatty acid in ostrich oil, constituting 34.60 ± 0.01% of the composition. Oleic acid is known for its antioxidant and antibacterial activities, particularly in inhibiting the growth of various Gram-positive bacterial species [51,52]. Consequently, the release study focused on oleic acid. The maximum percentage release of oleic acid from the optimized emulsion containing ostrich oil was 12.06 ± 1.05%, 28.34 ± 0.99%, 41.64 ± 1.06%, and 78.16 ± 1.22% at 30, 60, 90, and 180 min, respectively. In comparison, the release of ostrich oil alone was only 3.12% at 60 min, reaching 15.09% at 180 min. These findings demonstrate that the development of the optimized O/W emulsion containing ostrich oil significantly improved the rate of release across the cellulose acetate membrane, aligning with previous studies suggesting that emulsions enhance permeability [11].

This study revealed that the optimized emulsion containing ostrich oil improved the rate of release across the cellulose acetate membrane, potentially attributed to the presence of penetration enhancers such as Span 20 and Tween 80. Our investigation also indicated that as the concentration of the Span and Tween mixture increased, the droplet size of the optimized emulsion decreased. This reduction in droplet size (5.01 ± 0.43 μm) may have contributed to the improved kinetic release of ostrich oil across the membrane layer. This effect could be associated with the emulsifying properties of Tween 80, which lower interfacial tension and reduce droplet deformation [53].

## 3. Discussion

The heightened antioxidant and antibacterial characteristics observed in an emulsion containing ostrich oil, as opposed to ostrich oil alone, can be ascribed to a number of emulsification process-related factors and the synergistic influences of the emulsion constituents. There are several significant considerations. The emulsification process considerably increases the available surface area for interaction by reducing the ostrich oil to smaller droplets, thereby increasing the surface area available for interaction. The augmented surface area facilitates enhanced interaction between ostrich oil and reactive molecules or microorganisms, thereby augmenting the overall efficacy. In order to enhance dispersion, emulsification guarantees that the oil is distributed more uniformly across the formulation. The even dispersion of these compounds enhances the oil’s capacity to interact with microbes and free radicals, thereby optimizing its antibacterial and antioxidant properties [54]. Reduced droplet diameters within the emulsion have the potential to augment the bioavailability of bioactive compounds that are found in ostrich oil. The enhanced bioavailability of the oil’s beneficial components facilitates their improved absorption and utilization by epidermis cells or microbes [55]. In order to enhance the stability of the formulation, emulsions frequently incorporate emulsifiers and stabilizers (e.g., Span 20 and Tween 80) as stabilizing agents. Additionally, these agents might possess intrinsic qualities that augment their antimicrobial and antioxidant impacts as a whole [56]. The integration of emulsifiers with ostrich oil may result in synergistic effects, wherein the distinct constituents collaborate to augment the overall effectiveness. By forming a stable and adherent layer on the skin, emulsions can extend the duration of contact between the oil and the affected area or wound [57]. The protracted contact time facilitates a sustained release of bioactive compounds, thereby enhancing the duration of their antibacterial and antioxidant properties. As a result, the emulsification procedure serves to improve the chemical and physical characteristics of ostrich oils, thereby increasing their efficacy in demonstrating antioxidant and antibacterial properties. The enhanced properties of emulsions bearing ostrich oil can be attributed to a combination of factors, including increased surface area, improved dispersion, stabilizing agents, and possible synergistic effects.

## 4. Materials and Methods

### 4.1. Materials

All reagents utilized in the experiments were of analytical reagent (AR) and American Chemical Society (ACS) grade. Methyl heptadecanoate and all fatty acid GC standards were procured from Nu-Chek Prep, Inc., Elysian, MN, USA. The ICP multi-element standard solution XIII was acquired from Agilent Technologies, Santa Clara, CA, USA. Purchased from Sigma-Aldrich, St. Louis, MO, USA: 2,2-diphenyl-1-picrylhydrazyl (DPPH), 6-hydroxy-2,5,7,8-tetramethylchroman-2-carboxylic acid (Trolox), and Wijs solution. The reference standard, diclofenac diethylamine, was purchased from the Bureau of Drug and Narcotic, Department of Medical Sciences (DMSC), Ministry of Public Health (Nonthaburi, Thailand). Purchases were made of Mueller–Hinton agar (MHA), Tryptic Soy Broth (TSB), and Tryptic Soy Agar (TSA) from Sigma-Aldrich (St. Louis, MO, USA), Becton, Dickinson and Company (Sparks, MD, USA), and HiMedia Laboratories Private Limited (Mumbai, Maharashtra, India), respectively. Lincomycin was bought from T.P. Drug Laboratories (1969) Co., Ltd. (Phra Khanong, Bangkok, Thailand). Merck KGaA, Darmstadt, Germany supplied the Iscove’s Modified Dulbecco’s Media (IMDM) and 3-(4,5-dimethylthiazol-2-yl)-2,5-diphenyltetrazolium bromide (MTT).

### 4.2. Preparation and Evaluation of Ostrich Oil

#### 4.2.1. Preparation of Ostrich Oil

Ostrich frozen abdominal fat was bought from Siam Ostrich Farm, located in the Song Phi Nong district of Suphan Buri Province, Thailand. After thawing the ostrich fat samples at room temperature (25 °C), adherent tissues were removed, the samples were meticulously rinsed with distilled water, and any remaining surface moisture on the fat tissues was absorbed by tissue papers to remove excess water. Following that, the fat was further chopped into a smooth paste after being reduced to tiny pieces. The rendering method [1] was employed to extract the fat paste at a temperature of 50 °C, with continuous stirring until a complete melting of the fat occurred. After cooling to 25 °C, the resulting transparent oil underwent a cooling process, followed by a separation from the unmelted residues. To remove any remaining moisture, anhydrous sodium sulfate was added, and the oil was subsequently stored in nitrogen-filled amber glass vials at 4 °C. This precautionary measure was implemented to mitigate oxidation and photodegradation before its utilization in the formulation process.

#### 4.2.2. Fatty Acid Composition

As previously reported, fatty acid methyl esters (FAMEs) were produced using a one-step extraction and direct transesterification process between fatty acids and methanol at 90 °C for 30 min [1]. The solution of FAMEs was promptly analyzed using gas chromatography with a flame ionization detector (GC-FID) and an automated liquid sampler (Model 6890N Network GC System, Agilent Technologies, Santa Clara, CA, USA). Methyl heptadecanoate was used as an internal standard.

#### 4.2.3. Antioxidant Activity

The DPPH radical scavenging and lipid peroxidation inhibitory activities were conducted in line with our previous studies [1,58]. For the assessment of DPPH radical scavenging activities in the samples, 1 mL of the sample dissolved in ethyl acetate was introduced to a fresh solution of 0.1 mmol/L DPPH in ethyl acetate. Following incubation at 25 °C in a dark environment for 90 min, the absorbance of the solution was promptly measured at 517 nm using a UV-Vis spectrophotometer (Hitachi U-2900, Hitachi High-Technologies Corporation, Tokyo, Japan) against a blank. Trolox served as the chosen standard antioxidant. The determination of the 50 percent inhibitory concentration (IC_50_) was achieved through linear regression. The experiments were conducted in triplicate.

Lipid oxidation in a linoleic acid emulsion, employed as a model system to determine the antioxidant activities of samples, was assessed using an ammonium thiocyanate assay method [1], with some modifications. A 0.2 mol/L linoleic acid emulsion was meticulously prepared by precisely weighing linoleic acid (0.28 g) into a 50 mL volumetric flask and adjusting it with 0.56% (*w*/*v*) Tween 20 in 0.2 mol/L phosphate buffer solution (pH 7.0) to reach the final volume. For the experiment, 400 mg of the oil sample was blended with 5.0 mL of linoleic acid emulsion and 4.6 mL of phosphate buffer solution. The mixture was then incubated in a heat block at 37–40 °C in the dark for 60 min. Subsequently, 300 μL of each solution was extracted at day 0, 1, 2, 3, 4, 5, 6, and 7 for lipid oxidation measurements. To test for lipid peroxidation, the sampling solution was combined with 4.3 mL of 75% (*v*/*v*) ethanol, 0.2 mL of 30% (*w*/*v*) ammonium thiocyanate solution, and 0.2 mL of 0.2 mol/L ferrous chloride solution. After being left at room temperature for 3 min, the absorbance of the reaction solution was promptly measured at 500 nm against the corresponding blank using a UV-Vis Spectrophotometer (Hitachi U-2900, Hitachi High-Technologies Corporation, Tokyo, Japan). The control sample consisted of the same solution without any additives, while Trolox served as the standard antioxidant.

#### 4.2.4. Anti-Inflammatory Activity

The anti-inflammatory activity was evaluated by assessing the inhibition of heat-induced denaturation of egg albumin, following a modified version of the procedure outlined by Limmatvapirat et al. [59]. Concisely, the reaction mixture consisted of 0.2 mL of fresh egg albumin, 2.8 mL of phosphate-buffered saline (pH 7.4), and 2 mL of sample solution at concentrations ranging from 0.1 to 4 mg/mL. All of the mixtures were then incubated at 37 ± 1 °C for 15 min and heated at 70 ± 1 °C for 5 min. After cooling to 25 ± 1 °C, absorbance values were measured at 660 nm using a UV-Vis Spectrophotometer (Hitachi U-2900, Hitachi High-Technologies Corporation, Tokyo, Japan). A graph depicting the relationship between the sample concentrations and the percentage inhibition of albumin protein denaturation was constructed. The IC_50_ inhibitory concentration was determined through linear regression analysis. Negative and positive controls, comprising distilled water and diclofenac diethylamine, respectively, were included for comparison purposes.

#### 4.2.5. Acid Value (AV) and Peroxide Value (PV)

AV and PV were evaluated using a potentiometric auto-titrator, Titrino Plus 848 (Metrohm, Herisau, Switzerland), in accordance with the American Oil Chemists’ Society (AOCS) official procedures Cd 3d63 [60] and Cd 8b-90 [61].

#### 4.2.6. Heavy Metal Contents

The concentrations of heavy metals were determined using inductively coupled plasma-mass spectrometry (ICP-MS), as in our previous study [62]. All glassware and plastic bottles were cleaned by soaking them in a 20% *v*/*v* nitric acid solution for a minimum of 24 h and were rinsed several times with a 5% *v*/*v* nitric acid solution to remove surface contamination. An ICP multi-element standard solution XIII was diluted with a 5% *v*/*v* nitric acid solution at 5 different concentrations. For sample digestion, 1 g of each sample was mixed with 10 mL of a 60% *v*/*v* nitric acid solution and digested using a microwave digester (Model ETHOS ONE, Milestone Corporation, Sorisole, Italy). The digested sample solution was allowed to cool to 25 °C and then filtered. The digested filtrate was diluted to the appropriate concentration with ultra-pure water. The resulting solution was analyzed using an ICP-MS instrument (Agilent Technologies 7500 ce, Santa Clara, CA, USA). The operating conditions included an ICP radio frequency power of 1500 W, an ultra-pure-grade carrier (argon, 99.9995% pure) flow rate of 0.9 L/min, and a nebulizer pump speed of 0.1 round per second (rps). All of the samples were digested in triplicate, and the concentrations of heavy metals were calculated by determining the average of the triplicate measurements.

#### 4.2.7. Microbial Contamination

The total aerobic microbial count (TAMC), the total combined yeast and mold count (TYMC), and the individual results of *S. aureus*, *P. aeruginosa*, *Clostridium* spp., and *Candida albicans* in the samples were analyzed to determine the microbial contamination. All of the sample enumeration tests were conducted in accordance with the United States Pharmacopoeia 43 and National Formulary 38 [63]. To determine the TAMC and TYMC using the plate method, 1 mL of the diluted sample solution was inoculated into 20 mL of TSA and SDA, respectively. The inoculated plates were then incubated at 32.5 ± 0.5 °C for 5 days for TSA and 25 ± 0.5 °C for 7 days for SDA. The appropriate dilutions for inoculation were prepared within 1 h before adding the sample to the medium. After incubation, microbial colonies on the plates were counted as colony forming units (CFU) per gram of sample. For the cultivation of other specified microbes, including *S. aureus*, *P. aeruginosa*, *Clostridium* spp., and *C. albicans*, plates were incubated at 32.5 ± 0.5 °C for 24–48 h. The non-growing samples were plated onto selective agars and incubated at 32.5 ± 0.5 °C for 24–72 h to examine growth.

### 4.3. Formulation and Evaluation of Emulsion Containing Ostrich Oil

#### 4.3.1. Formulation of O/W Emulsion

- Determination of the required hydrophile-lipophile balance (HLB) for emulsification

As depicted in Table 2, O/W emulsions were formulated with 20% *w*/*w* ostrich oil and 5% *w*/*w* emulsifiers. The emulsifiers, a blend of sorbitan monooleate (Span 80, HLB 4.3) and polyoxyethylene (20) sorbitan monooleate (Tween 80, HLB 15.0), were used in various ratios calculated based on the total emulsifier content. To ascertain the HLB values, the emulsions were prepared using the beaker method, which was slightly modified from the previous procedure [23,24,25]. Tween 80 was dissolved in the aqueous phase, while Span 80 was dissolved in the oil phase. At 65 °C and 62 °C, the aqueous phase and the oil phase were heated, respectively. Slowly pouring the oil phase into the water phase, this emulsion was then homogenized at 3800 rpm for 5 min using a T25 digital Ultra-Turrax homogenizer (IKA, Baden-Württemberg, Germany). Further evaluation was conducted on the physicochemical properties of all emulsions with HLB values between 4.3 and 8.0.

The homogenization speed in the preparation of the O/W emulsion is a crucial parameter with a significant impact on the emulsion’s characteristics. The selection of homogenization speed is grounded in theoretical principles supporting the notion that higher speeds can yield smaller emulsion droplets. Smaller droplets enhance stability by providing a larger interfacial area for emulsifiers to act upon. Elevated homogenization speeds generate more intense shear forces, breaking down larger oil droplets into smaller ones. This reduction in droplet size contributes to improved stability by preventing coalescence and promoting homogeneity in the system [64]. However, the homogenization speed must be carefully balanced with other factors, including the type and concentration of emulsifiers, the nature of the oil phase, and the desired characteristics of the final emulsion. The choice of 3800 rpm for the homogenization speed in the preparation of the ostrich oil-based O/W emulsion was determined through preliminary experiments, striking a balance between achieving the desired droplet size for stability and practical considerations, such as equipment limitations.

- Effects of the type and concentration of mixed emulsifiers on the properties of emulsions

Utilizing Span and Tween as emulsifiers, ostrich oil emulsions were created. The effects of the different emulsifier types, including sorbitan monolaurate (Span 20, HLB 8.6), Span 80 (HLB 4.3), polyoxyethylene (20) sorbitan monolaurate (Tween 20, HLB 16.7), polyoxyethylene (20) sorbitan monostearate (Tween 60, HLB 14.9), and Tween 80 (HLB 15.0), on the properties of O/W emulsions were comparatively evaluated. Also evaluated were the effects of varying concentrations of combined emulsifiers (Span and Tween) at 5, 10, 15, and 20% *w*/*w*. According to the experiment described above, each emulsion was prepared using a particular combination of emulsifiers to achieve the required HLB. The oil phase was produced by adding Span to 20% *w*/*w* ostrich oil and stirring the mixture at 62 °C with a magnetic stirrer. The aqueous phase was created by dissolving a specific volume of Tween in distilled water at 65 °C, and then the oil phase was continuously poured into the aqueous phase. This emulsion was homogenized for 5 min at 3800 rpm with a homogenizer. The obtained emulsions were then evaluated.

#### 4.3.2. Evaluation of Emulsion

By means of visual observation, the physical properties and creaming indices of emulsions were ascertained. The creaming index was calculated utilizing Equation (1), in which S denotes the serum layer’s height and T signifies the emulsion’s total height:

(1) $$\%\mathrm{Creaming}\;\mathrm{index}=\frac{S}{T}\times 100$$

The size of the oil droplets in the emulsion was determined using a laser scattering particle-size-distribution analyzer model LA950 (Horiba Ltd., Kyoto, Japan). The zeta potential value was measured using a ZetaPlus zeta potential analyzer (Brookhaven Instruments Corporation, New York, NY, USA). A Brookfield DV-III Ultra Programmable rheometer, model RVDV-III Ultra (Brookfield Engineering Laboratories, Inc., Middleborough, MA, USA), equipped with a CPE-51 or CPE-40 spindle, was utilized to determine the viscosity at a temperature of 25 °C. The morphology of the oil droplets was examined using an Olympus CX41RF optical microscope (Olympus Corporation, Tokyo, Japan).

#### 4.3.3. Stability Test

Samples from the optimized emulsion, consisting of 20% *w*/*w* ostrich oil, 15% *w*/*w* mixed emulsifiers (Span 20 and Tween 80), both with and without 0.01% *w*/*w* butylated hydroxytoluene (BHT), underwent evaluation for AV, PV, phase separation, and microbial contamination. These stability assessments were conducted using amber vials sealed with hermetically sealed lids, shielded from light, and maintained at temperatures of 4 ± 0.5 °C, room temperature (25 ± 0.5 °C), and 45 ± 0.5 °C. The analysis intervals included the initial, 1st, 3rd, and 6th months. Control analyses were performed on the fresh samples (time zero). To mitigate potential variations caused by different storage conditions, all of the collected emulsion and ostrich oil samples at each time duration were promptly analyzed.

#### 4.3.4. Cytotoxicity Assay

In order to evaluate the cytotoxicity of the optimized formulation on human dermal fibroblasts, ATCC^®^ CRL-2076 (Manassas, VA, USA), the MTT colorimetric assay [65] was utilized. To attain the intended concentration range, IMDM was utilized to appropriately dilute each sample. In a 96-microwell plate, cells were inoculated at a density of 1 × 10^4^ cells/well and cultured until they achieved a confluency of 80–90%. The cells were subsequently exposed to 100 μL of sample-containing media at 37 °C in an incubator containing 5% CO_2_ for 24 h. After adding 10 µL of a 5 mg/mL MTT solution to each well, the mixture was incubated for an additional 2 h. Following the removal of the supernatant, 100 µL of dimethyl sulfoxide (DMSO) were introduced in order to solubilize the formazan products generated by viable cell mitochondria. The absorbance at 550 nm was determined using a Fusion universal microplate analyzer (Model A153601, Packard BioScience Company, Meriden, CT, USA). In comparison to untreated controls, the percentages of viable cells were calculated.

#### 4.3.5. Antibacterial Assay

The antibacterial activity of the optimized emulsion was evaluated using the agar disc diffusion technique against three bacterial strains: *S. aureus* ATCC 6538P, *Escherichia coli* DMST 4212, and *P. aeruginosa* ATCC 9027 [66]. The McFarland standard number 0.5 was prepared by mixing 9.95 mL of 1% *w*/*v* sulfuric acid with 0.05 mL of 1.175% *w*/*v* barium chloride dihydrate to estimate bacterial density. The inoculum of each test bacterium was prepared in sterile MHA, and the turbidity of each suspension was adjusted to match the 0.5 MacFarland standard, equivalent to 1.5 × 10^8^ CFU/mL for bacteria using dilution method. Subsequently, 1.5 × 10^8^ CFU/mL of each test strain were inoculated onto MHA plates, which were then allowed to dry. A volume of 30 µL of the emulsion sample was added to sterile paper discs, each with a diameter of 6 mm. Subsequently, the saturated paper discs were dispensed onto the inoculated agar plate. Following the incubation of the plates at 37 °C for 18–24 h, inhibitory zones (including the 6 mm disc diameter) were determined. To determine whether the emulsion sample was as potent as the commercial antibacterial lincomycin, a parallel analysis study was performed with it at 30 µg/disc. A control emulsion containing a placebo was utilized. Each and every test was performed in triplicate.

#### 4.3.6. In Vitro Release Study

In this research study, in vitro release investigations were conducted utilizing Franz diffusion cells (PermeGear, Hellertown, PA, USA), following a previously established protocol [67]. A cellulose acetate membrane with a pore size of 0.2 µm (Whatman 10404112, Cellulose Acetate Membrane Circle, Merck KGaA, Darmstadt, Germany) was employed to facilitate the differentiation of released substances, positioned between the donor and receptor compartments. In each trial, 0.1 mL of the optimized emulsion was uniformly applied to the membrane. The receptor compartments were filled with a phosphate buffer (pH 7.4) as the dissolution medium, agitated with a magnetic stir bar, and maintained at 37 °C. At 0, 30, 60, 90, and 180 min subsequent to emulsion application, 2 mL of the receptor medium was withdrawn and replaced with an equivalent volume of fresh phosphate buffer to maintain a constant volume (5 mL) in the lower chamber. Each withdrawn solution underwent triple partitioning with hexane to extract the released substances. The hexane layers obtained from each extraction were combined. Fatty acids within the hexane extract solution underwent conversion into FAMEs, utilizing a one-step extraction and direct transesterification process with methanol at 90 °C for 30 min [1]. The FAMEs solution was subjected to analysis using GC-FID (Model 6890N Network GC System, Agilent Technologies, Santa Clara, CA, USA), with methyl heptadecanoate employed as an internal standard. The percentage release was calculated using a standard curve. All experiments were conducted in triplicate, and the outcomes are presented as mean values. Statistical analysis was carried out to interpret the data.

### 4.4. Statistical Analysis

The data were statistically analyzed using the *t*-test and one-way analysis of variance (ANOVA) (version 16 of the SPSS program) on measurements performed in triplicate. *p*-values less than 0.05 were statistically significant.

## 5. Conclusions

The objective of this research was to develop an O/W emulsion, optimized by incorporating a substantial 20% ostrich oil and 15% *w*/*w* blended emulsifiers (Span 20 and Tween 80) via homogenization. The optimized O/W emulsion exhibited a particle size of 5.01 ± 0.43 μm and a zeta potential of −32.22 mV, indicative of its stability and potential for topical administration. Safety testing on CRL-2076 human dermal fibroblasts revealed a positive safety profile. Enriched with ostrich oil, the optimized O/W emulsion demonstrated significantly heightened antibacterial and antioxidant properties compared to ostrich oil alone. Notably, the emulsion facilitated the release of 78.16 ± 1.22% of oleic acid across the cellulose acetate membrane after 180 min of study time, indicating effective diffusion of this bioactive compound with known antioxidant and antibacterial properties. These findings underscore the potential of the optimized emulsion as a delivery system for bioactive compounds, with implications for transdermal administration and topical treatments. Stability experiments conducted over 6 months at temperatures of 4 °C, 25 °C, and 45 °C confirmed the physical and microbial stability of the emulsion, further supporting its viability for practical applications. This research highlights the feasibility of employing ostrich oil in topical formulations and emphasizes its efficacy in O/W emulsions. The promising results suggest potential uses in the formulation of topical emulsions incorporating various animal oils, opening avenues for further exploration in the fields of skincare and pharmaceutical development.

## Figures and Tables

**Table 1:** Retention time and linearity of FAME standards.

FAME Standards	Retention Time (min)	Regression Equation (y = ax + b)	Correlation Coefficient (r^2^)
Methyl laurate	2.8	y = 1.0931x + 0.0013	0.9998
Methyl myristate	4.1	y = 1.1781x + 0.0023	0.9998
Methyl palmitate	6.8	y = 0.8441x − 0.0100	0.9998
Methyl stearate	12.2	y = 0.9189x − 0.0121	0.9998
Methyl oleate	13.1	y = 1.2001x − 0.0087	0.9999
Methyl linoleate	15.1	y = 1.1643x + 0.0116	0.9998
Methyl linolenate	18.5	y = 1.1147x − 0.0014	0.9998

**Table 2:** O/W emulsions containing 20% *w*/*w* ostrich oil and 5% *w*/*w* mixed emulsifiers (Span 80 and Tween 80), as well as their required HLB values and creaming indices.

Formulation	Distilled Water (% *w*/*w*)	Ostrich Oil (% *w*/*w*)	Emulsifier (5% *w*/*w*)			HLB	Creaming Index (%)
			Span 80 (% *w*/*w*)	Tween 80 (% *w*/*w*)	Span 80:Tween 80 (*w*/*w*)		
1	75	20	5	0	5:0	4.3	12.38
2	75	20	4.82	0.18	26:1	4.7	11.29
3	75	20	4.67	0.33	14:1	5.0	4.27
4	75	20	4.44	0.56	8:1	5.5	0.00
5	75	20	4.20	0.80	5:1	6.0	9.69

**Table 3:** O/W emulsion with an HLB of 5.5 containing 20% *w*/*w* ostrich oil and 5, 10, 15, or 20% *w*/*w* combined Span (Sp) and Tween (Tw) emulsifiers.

Fomulation	Distilled Water (% *w*/*w*)	Ostrich Oil (% *w*/*w*)	Span (Sp) (% *w*/*w*)	Tween (Tw) (% *w*/*w*)
5% *w*/*w* mixed emulsifiers				
Sp80Tw80	75	20	4.44	0.56
Sp80Tw60	75	20	4.42	0.58
Sp80Tw20	75	20	4.52	0.48
Sp20Tw80	75	20	3.72	1.28
Sp20Tw60	75	20	3.70	1.30
Sp20Tw20	75	20	3.76	1.24
10% *w*/*w* mixed emulsifiers				
Sp80Tw80	70	20	8.88	1.12
Sp80Tw60	70	20	8.86	1.14
Sp80Tw20	70	20	9.04	0.96
Sp20Tw80	70	20	7.54	2.46
Sp20Tw60	70	20	7.52	2.48
Sp20Tw20	70	20	7.84	2.16
15% *w*/*w* mixed emulsifiers				
Sp80Tw80	65	20	13.32	1.68
Sp80Tw60	65	20	13.30	1.70
Sp80Tw20	65	20	13.54	1.46
Sp20Tw80	65	20	11.30	3.70
Sp20Tw60	65	20	11.28	3.72
Sp20Tw20	65	20	11.74	3.26
20% *w*/*w* mixed emulsifiers				
Sp80Tw80	60	20	17.76	2.24
Sp80Tw60	60	20	17.74	2.26
Sp80Tw20	60	20	18.06	1.94
Sp20Tw80	60	20	15.08	4.52
Sp20Tw60	60	20	15.04	4.96
Sp20Tw20	60	20	15.66	4.34

**Table 4:** Creaming index percentages of emulsions containing 20% *w*/*w* ostrich oil and a 5, 10, 15, or 20% *w*/*w* mixture of Span (Sp) and Tween (Tw) on days 1, 3, and 7.

Formulation	Percent of Creaming Index		
	Day 1	Day 3	Day 7
5% *w*/*w* mixed emulsifiers			
Sp80Tw80	0.00	11.32	25.45
Sp80Tw60	30.48	34.55	45.45
Sp80Tw20	7.41	20.00	23.64
Sp20Tw80	50.91	60.00	60.00
Sp20Tw60	46.73	50.91	54.55
Sp20Tw20	46.73	50.91	54.55
10% *w*/*w* mixed emulsifiers			
Sp80Tw80	0.00	5.56	23.64
Sp80Tw60	0.00	7.41	36.36
Sp80Tw20	0.00	7.41	35.71
Sp20Tw80	40.00	41.07	52.63
Sp20Tw60	51.79	52.63	61.02
Sp20Tw20	35.71	35.09	51.67
15% *w*/*w* mixed emulsifiers			
Sp80Tw80	0.00	7.14	18.97
Sp80Tw60	0.00	9.09	36.84
Sp80Tw20	0.00	7.14	44.83
Sp20Tw80	0.00	0.00	0.00
Sp20Tw60	0.00	3.45	8.20
Sp20Tw20	0.00	0.00	0.00
20% *w*/*w* mixed emulsifiers			
Sp80Tw80	0.00	0.00	0.00
Sp80Tw60	0.00	0.00	6.52
Sp80Tw20	0.00	0.00	6.52
Sp20Tw80	0.00	0.00	0.00
Sp20Tw60	0.00	0.00	0.00
Sp20Tw20	0.00	0.00	0.00

**Table 5:** AVs and PVs of the optimized emulsion with and without BHT.

Sample	Storage Temperature (°C)	AV (mg KOH/g Sample)				PV (mEq O_2_/kg Sample)			
		Initial	1 Month	3 Months	6 Months	Initial	1 Month	3 Months	6 Months
Optimized emulsion with BHT	4 °C	0.09 ± 0.01	0.12 ± 0.01	0.12 ± 0.01	0.12 ± 0.01	2.50 ± 0.10	2.50 ± 0.10	2.50 ± 0.11	2.50 ± 0.10
	25 °C		0.12 ± 0.01	0.12 ± 0.01	0.12 ± 0.01		2.50 ± 0.10	2.61 ± 0.12	2.61 ± 0.11
	45 °C		0.13 ± 0.01	0.14 ± 0.01	0.23 ± 0.02		2.55 ± 0.10	2.98 ± 0.15	3.72 ± 0.21
Optimized emulsion without BHT	4 °C	0.09 ± 0.01	0.12 ± 0.01	0.12 ± 0.01	0.12 ± 0.01	2.50 ± 0.10	2.51 ± 0.10	2.95 ± 0.13	3.30 ± 0.20
	25 °C		0.13 ± 0.01	0.14 ± 0.02	0.17 ± 0.02		2.51 ± 0.11	2.98 ± 0.12	4.51 ± 0.21
	45 °C		0.14 ± 0.02	0.16 ± 0.02	0.32 ± 0.02		3.31 ± 0.11	5.61 ± 0.19	7.51 ± 0.25
Ostrich oil	4 °C	0.09 ± 0.01	0.12 ± 0.01	0.12 ± 0.01	0.12 ± 0.01	2.50 ± 0.10	2.51 ± 0.10	2.95 ± 0.13	3.30 ± 0.20
	25 °C		0.13 ± 0.01	0.14 ± 0.02	0.18 ± 0.01		2.55 ± 0.13	3.18 ± 0.10	4.89 ± 0.15
	45 °C		0.14 ± 0.02	0.17 ± 0.01	0.34 ± 0.02		3.50 ± 0.14	5.80 ± 0.19	8.91 ± 0.20

**Table 6:** Antibacterial and antioxidant activities of the ostrich oil and its optimized emulsion.

Samples	Inhibition Zone (mm)			DPPH IC_50_ (mg/mL)	Linoleic Acid Inhibition (%), Day 5
	*S. aureus*	*E. coli*	*P. aeruginosa*		
Optimized emulsion	12.32 ± 0.19 ^a^	6.93 ± 0.18 ^a^	6.3 ± 0.14 ^a^	23.52 ± 1.09 ^b^	52.20 ± 2.01 ^a^
Ostrich oil	6.12 ± 0.15 ^b^	0 ^b^	0 ^b^	39.92 ± 1.51 ^a^	44.70 ± 1.94 ^b^
Lincomycin	27.00 ± 0.65	21.03 ± 0.25	8.02 ± 0.18	NA	NA
Trolox	NA	NA	NA	0.0043 ± 0.0001	58.20 ± 5.18

The data are the means of three independent experiments ± standard deviations (*n* = 3). ^a, b^ Values in the same column with different superscript letters differ significantly (*p* < 0.05). Zero, no inhibition; NA, not applicable.

## Data Availability

Upon request, the corresponding author will provide access to the data utilized to substantiate the conclusions drawn in this study.
